# Antibacterial and Antifungal Activities of *Cimbopogon winterianus* and *Origanum syriacum* Extracts and Essential Oils against Uropathogenic Bacteria and Foodborne Fungal Isolates

**DOI:** 10.3390/foods13111684

**Published:** 2024-05-27

**Authors:** Marwa Rammal, Salam Khreiss, Adnan Badran, Malak Mezher, Mikhael Bechelany, Chaden Haidar, Mahmoud I. Khalil, Elias Baydoun, Mohammad H. El-Dakdouki

**Affiliations:** 1 Department of Food Sciences and Technology, Faculty of Agronomy, Lebanese University, Beirut P.O. Box 146404, Lebanon; marwa.rammal.1@ul.edu.lb (M.R.); salam.khreiss@st.ul.edu.lb (S.K.); chaden.haidar@iul.edu.lb (C.H.); 2 Department of Nutrition, University of Petra, Amman P.O Box 961343, Jordan; abadran@uop.edu.jo; 3 Department of Biological Sciences, Faculty of Science, Beirut Arab University, P.O. Box 11-5020, Beirut 11072809, Lebanon; mezher.malak@outlook.com (M.M.); or m.khalil@bau.edu.lb (M.I.K.); 4 Institut Européen des Membranes (IEM), UMR-5635, Université de Montpellier, École Nationale Supérieure de Chimie de Montpellier (ENSCM), Centre National de la Recherche Scientifique (CNRS), Place Eugene Bataillon, 34095 Montpellier, France; 5 Functional Materials Group, Gulf University for Science and Technology (GUST), Mubarak Al-Abdullah 32093, Kuwait; 6 Molecular Biology Unit, Department of Zoology, Faculty of Science, Alexandria University, Alexandria 21568, Egypt; 7 Department of Biology, American University of Beirut, P.O. Box 11-0236, Beirut 11072020, Lebanon; eliasbay@aub.edu.lb; 8 Department of Chemistry, Faculty of Science, Beirut Arab University, Riad El Solh, P.O. Box 11-5020, Beirut 11072809, Lebanon

**Keywords:** *O. syriacum*, *C. winterianus*, extracts, essential oils, antibacterial activity, antifungal activity, preservation, food packaging

## Abstract

This study focused on testing the antibacterial and antifungal activity of *Origanum syriacum* (*O. syriacum*) and *Cimbopogon winterianus* (*C. winterianus*) extracts and their essential oils (EOs). The bacteria were isolated from urine samples and identified by a VITEK assay, and the fungi were isolated from spoiled food samples and further identified by MALDI-TOF. The susceptibility of the microbial isolates was assessed by determining the bacteriostatic and bactericidal/fungicidal effects by the minimum inhibitory concentration (MIC) and minimum bactericidal/fungicidal concentration (MBC/MFC) broth microdilution assay and time-kill test. The antibiofilm activities were assessed by the antibiofilm screening assays. The bacterial isolates included three Gram-negative isolates (*Escherichia coli*, *Klebsiella pneumonia*, and *Citrobacter freundii*) and two Gram-positive isolates (*Staphylococcus aureus* and *Streptococcus intermedius*). The fungal isolates included *Candida albicans* and *Aspergillus niger*. The *O. syriacum* and *C. winterianus* extracts exhibited bacteriostatic and fungistatic activities (MIC 1.25–2.5 mg/mL for the bacterial isolates and 2.5–5 mg/mL for the fungal isolates). However, their EOs exhibited bactericidal (MBC 5–20%) and fungicidal (MFC 1.25–10%) activities, meaning that the EOs had a better antimicrobial potential than the extracts. The antibiofilm activities of the mentioned extracts and their EOs were relatively weak. The *O. syriacum* extract inhibited *S. aureus*, *S. intermedius*, and *K. pneumonia* biofilms at a concentration of 0.3125 mg/mL and *C. albicans* and *A. niger* biofilms at 0.625 mg/mL. No antibiofilm activity was recorded for *C. winterianus* extract. In addition, the packaging of grapes with *C. winterianus* extract preserved them for about 40 days. The results reflect the significant antimicrobial activity of *O. syriacum* and *C. winterianus* extracts and their EOs, thus suggesting their potential in food packaging and preservation.

## 1. Introduction

Food packaging faces significant economic, environmental, and public health challenges. It is time to mobilize all stakeholders to advance materials and technologies toward solutions that are more friendly to the environment and respectful to the consumer [1]. Usually, food packages are made of plastic. They are essential for food because they provide protection against the negative influences of the outer environment [2]. For this reason, consumers have started looking for alternative food packaging materials due to the consumer demand for non-synthetic products and the growing concerns about plastic’s effects on the environment [2,3].

Plastic can be replaced with biodegradable materials like biopolymers, bioplastics, bio-nanocomposites, and edible coatings [3]. Healthy alternatives to traditional food packaging are edible films and coatings. This is why research has shed light on forming biopolymer-based biodegradable packaging materials [4]. Aliphatic polysaccharides, proteins, and polyesters are the main biopolymers applied in food preservation because they increase the shelf life of products. These packaging materials act as barriers which control gas, humidity, aromas, and lipids exchange with the outside environment [2,5]. They possess antimicrobial activities, thus protecting the food products against the external environment and preventing the loss of flavors, texture, and other desirable elements [2,5,6,7].

Natural polymers from plants or animals have been used by several researchers [2]. Biopolymers are activated by additives including antimicrobials, antioxidants, nutrients, essential oils (EOs), phenolic compounds, and plant extracts. This improves the food’s shelf life. For example, adding glycerol and sorbitol to biopolymers can modify their brittleness, transformability, starch chain, mobility, and moisture absorption [3].

Grape fruit is non-climacteric and perishable after harvest. It begins to deteriorate due to the loss of water, fungal rot, and oxidation. This causes rachis blackening and turgor loss, thus affecting the characteristics of the grape product and making it unsaleable [8,9]. Edible coatings (EDCs) have been widely used as preservatives for fresh fruits because they extend their post-harvest shelf life [8,9]. EDCs form thin layers of macromolecules that are applied to food surfaces, where they act as semi-permeable barriers to water vapor and gases. This reduces the respiration of fruits and in turn the loss of weight [8,10]. In addition, EDCs maintain fruit firmness and give shine to the coated product [8,11]. They can be made from various polysaccharides. For example, starch is a natural polysaccharide which has a high molecular weight and is inexpensive, poorly degradable, and well biocompatible [12].

EOs are hydrophobic liquids made up of volatile aromatic compounds that have a variety of effects associated with their antimicrobial, anti-inflammatory, antioxidant, and anticancer effects like the EO of *O. syriacum* [13]. At the same time, the EO of *C. winterianus* has several therapeutic applications. It is used as a mosquito repellent and as an antiparasitic, nematocidal, antifungal, and antibacterial agent [12]. The antimicrobial effect of extracts and EOs is usually caused by phenolics and monoterpenes, especially carvacrol and thymol. Carvacrol is considered one of the most effective antimicrobials [14]. The effectiveness of *O. syriacum* extract against a wide spectrum of pathogenic microorganisms has been reported [14,15]. In general, the effectiveness is linked to the high carvacrol content in the extract. The high lipophilic content of *O. syriacum* EO was linked to significant antibacterial activity against Gram-negative bacteria, while the high thymol content was more potent against Gram-positive bacteria. Both thymol and carvacrol are involved in the disruption of the bacterial cell membrane and the inhibition of ATPase activity [15]. In addition, the Lebanese Za’atar volatile EO showed inhibitory effects on fungi, especially *Penicillium*, *Aspergillus*, and *Fusarium*. It acts by inhibiting mycelial growth [16].

The roots and leaves of *C. winterianus* EOs are shown to have variable antimicrobial activities. The EO extracted from *C. winterianus* leaves exhibited potent activity against *Pseudomonas aeruginosa*, *Streptococcus pyogenes*, *Staphylococcus aureus*, and *Staphylococcus epidermidis*. This effect could be due to the individual components present in the EO and the synergistic mechanism between the EO components. Furthermore, the EOs extracted from *C. winterianus* leaves and roots exhibited potent antifungal activity against *Aspergillus fumigatus*, *Aspergillus niger*, *Trichophyton mentagrophytes*, *Microsporumcanis*, *Candida albicans*, and *Trichophyton mentagrophytes*. This effect could be due to the synergistic effect of the components of the EO [17].

At the end of the last century, research shifted from biodegradable film to fully degradable biofilms [18]. Various groups of organic antimicrobial agents are used in the packaging industry, such as antimicrobial peptides, organic acids, enzymes, and agents of natural plant origin. The function of biodegradable packaging film is the same as that of conventional packaging, which is to protect the food quality and increase its value. To date, molecules used to assemble fully biodegradable films include polysaccharides, such as starch [18]. Coatings can be applied to foods by various methods, including spraying, dipping, spreading, and thin-film hydration [3].

Few studies have explored the antimicrobial activity of *Oregano* and *Cymbopogon*. In this regard, the present work aims to study the antibacterial, antifungal, and antibiofilm activities of the combined *O. syriacum* and *C. winterianus* extracts and EOs against five uropathogenic bacteria (*Escherichia coli*, *Klebsiella pneumonia*, *Citrobacter freundii*, *Staphylococcus aureus*, and *Streptococcus intermedius*) and two foodborne fungi (*Candida albicans* and *Aspergillus niger*). In addition, it aims to develop a biodegradable film from biopolymers, starch and glycerol, and bioactive substances extracted from *C. winterianus* in order to preserve grapes after harvesting.

## 2. Materials and Methods

### 2.1. Isolation and Identification of the Bacterial and Fungal Isolates

Five uropathogenic bacteria were used to test the antibacterial potential of the aqueous and ethanolic *O. syriacum* and *C. winterianus* extracts, as well as their EOs. The bacterial isolates included three Gram-negative bacteria (*Escherichia coli*, *Klebsiella pneumonia*, and *Citrobacter freundii*) and two Gram-positive bacteria (*Staphylococcus aureus* and *Streptococcus intermedius*). All bacteria were isolated from urine. The isolation was performed by spreading 100 µL of the urine samples on various selective media. Following that, Gram staining was applied to separate the isolates into Gram-positive and Gram-negative isolates. The bacteria were further identified by VITEK (VITEK System 8.02 Version; Shenzhen, China). The VITEK assay relies on preparing a bacterial suspension in sodium chloride (NaCl; DB09153, Matangi, Honduras), adjusting it to 0.5 McFarland, and inserting it into VITEK cards for biochemical tests. The levels of identification are classified as excellent (96–99%), very good (93–95%), good (89–92%), and acceptable (85–88%). As for the fungal isolates, two fungi (*Candida albicans* and *Aspergillus niger*) were used. These fungi were isolated from food samples (lemon and jam) by spreading 100 µL of the samples on a specific medium (Potato Dextrose agar (PDA); HiMedia Laboratories Pvt. Ltd., Thane, India) and further identified by matrix-assisted laser desorption ionization–time of flight mass spectrometry (MALDI-TOF) (Autof MS1000 MALDI-TOF MS; Senegal, Africa) [19]. All bacteria and fungi were obtained and isolated in the Advanced Microbiology laboratory at Beirut Arab University.

### 2.2. Preparation of the Aqueous and Ethanolic O. syriacum and C. winterianus Extracts and EOs

The aqueous and ethanolic powdered *O. syriacum* and *C. winterianus* extracts were dissolved in each of their solvents (water and ethanol, respectively) to prepare solutions ranging in concentration between 0.3125 and 5 mg/mL. The EOs were dissolved in 1% dimethyl sulfoxide (DMSO; Inc. P.O. Box 439, Ghent, Belgium, KY 410455,1-800/367-6935) to prepare solutions of concentrations ranging between 1.25 and 20% (v:v) [19]. The aqueous *O. syriacum* and *C. winterianus* extracts were combined by mixing equal masses of the powdered extracts in distilled water to form concentrations ranging between 0.3125 and 5 mg/mL. The same preparation was performed for the ethanolic extracts. The EOs were prepared according to Rammal et al. [20] by hydro-distillation. Briefly, the leaves were heated with distilled water and the vapor was condensed to separate the oils from water. The EOs were stored at 4 °C. The *O. syriacum* and *C. winterianus* EOs were combined by mixing equal volumes of the EOs and dissolving them in DMSO to prepare solutions of concentrations ranging between 1.25 and 20%. In addition, the chemical composition of the EOs was tested by Gas Chromatography (GC; Biobase, BK-GC7820, Wolfenbuttel, Germany) as described by Rammal et al. [20] using an Agilent 6890N network gas chromatograph equipped with an Agilent 19091S-433HP-5MS column, with dimensions of 30 m × 0.25 mm × 0.25 μm. Briefly, the oven temperature was programmed to increase from 65 °C to 450 °C at a rate of 3 °C per min. Subsequently, detector scanning was conducted for a duration of 45 min. The identification of the reported individual components was achieved through a comparison of the mass spectra and by referring to the GC-MS library.

### 2.3. Minimum Inhibitory Concentration (MIC) and Minimum Bactericidal/Fungicidal (MBC/MFC) of the Aqueous and Ethanolic O. syriacum and C. winterianus Extracts and EOs by the Microdilution Assay

The MICs and MBCs of the aqueous and ethanolic *O. syriacum* and *C. winterianus* extracts and EOs were determined against all bacterial and fungal isolates by employing the micro-well dilution assay. The bacterial isolates were implanted into Muller Hinton broth (MHB; OXOID Ltd., Basingstoke, UK) until the turbidity matched the 0.5 McFarland scale. The fungal isolates were implanted into Potato Dextrose broth (PDB, HiMedia Laboratories Pvt. Ltd., Thane, India) (optical density = 0.08–0.1 at 600 nm). The test was carried out in sterile 96-well microplates by adding 90 µL of MHB or PDB and 10 µL of the bacterial and fungal suspensions into each well. Then, 100 µL of the prepared extracts (ranging between 0.3125 and 5 mg/mL) and their EOs (ranging between 1.25 and 20%) were added to the wells. Doxycycline (Dox; 100 mg, Actavis, Barnstaple, EX32 8NS, UK) was used as a reference antibiotic against the bacterial isolates, and cultures of bacteria and fungi without any treatment were used as negative controls. The plates containing bacteria were incubated for 24 h at 37 °C, and the plates containing fungi were incubated for 5 days at 30 °C. Optical density (O.D.) was measured at 595 nm (ELISA microtiter plate reader, Thermo Fisher Scientific, Shanghai, China), and the MIC was established as the lowest concentration of extract or EO that inhibited visible growth of bacteria and fungi. The MBCs/MFCs were determined by inoculating a loop-full of the clear wells into Muller Hinton agar (MHA; HiMedia Laboratories, Thane, India) plates and incubating them at 37 °C for 24 h (bacteria) and 30 °C for 5 days (fungi). The lowest concentration not exhibiting bacterial or fungal growth was recorded as the MBC/MFC. All experiments were performed in triplicate [19].

### 2.4. Determination of the Time Needed by the Aqueous and Ethanolic O. syriacum and C. winterianus Extracts and EOs to Inhibit Bacterial and Fungal Growth by the Time-Kill Assay

The time-kill assay was used to test the time needed by the aqueous and ethanolic *O. syriacum* and *C. winterianus* extracts and their EOs to exert their inhibitory activity against the bacterial and fungal isolates. The test was performed in 96-well microplates by adding 90 µL of MHB and 10 µL of bacterial suspensions adjusted to 0.5 McFarland and 90 µL of PDB and 10 µL of fungal suspensions (O.D. = 0.08–0.1 at 600 nm). Then, 100 µL of the MICs of the aqueous and ethanolic *O. syriacum* and *C. winterianus* extracts, as well as their EOs, were added into the 96-well microplates. The plates were incubated at 37 °C for bacterial isolates and 30 °C for fungal isolates, and the O.D. of bacterial growth was measured at 595 nm within the time interval (0–24 h), and that of fungal growth was measured within the time interval (1–5 days). The experiment was repeated at least three times [21,22].

### 2.5. Detection of the Anti-Biofilm Activity of the Aqueous and Ethanolic O. syriacum and C. winterianus Extracts and EOs by the Anti-Biofilm Assay

In order to detect the potential of the aqueous and ethanolic *O. syriacum* and *C. winterianus* extracts and EOs to prevent biofilm formation, the anti-biofilm formation test was applied. Bacteria and fungi were incubated in 96-well microtiter plates by adding 10 µL of standard bacterial or fungal suspension and 90 µL of MHB or PDB into each well. The plates were incubated at 37 °C (bacteria) and at 30 °C (fungi) for 30 min. After that, the aqueous and ethanolic *O. syriacum* and *C. winterianus* extracts and their EOs were added to the wells at different concentrations (extracts ranging between 0.3125 and 5 mg/mL and oils between 1.25 and 20%). Dox served as a reference antibiotic (positive control), and a bacterial culture without any treatment served as a negative control. The plates were incubated at 37 °C (bacteria) for 24 h and at 30 °C (fungi) for 5 days prior to being washed five times with sterile distilled water and oven dried (15 min at 60 °C). Then, 100 µL of 1% crystal violet (CV; 100 g, SURECHEM PRODUCTS Ltd., C8062; Paris, France) was added to stain the wells. The plates were incubated for 15 min at room temperature. The plates were then rinsed five times with sterile distilled water to remove the unabsorbed stain. The biofilms were noticeable as purple rings on the sides of the wells. Then, 100 µL of 95% ethanol (Dyadic international, Inc. OTCQX: DYAI, Jupiter, FL, USA) was added to the wells to destain them. An ELISA reader was used to detect the absorbance at 595 nm. The % inhibition of biofilm formation was calculated using the following equation:

$$\%\;\mathrm{Inhibition}=\frac{O.D.\left(negativecontrol\right)-O.D.(treatedsample)}{O.D.(negativecontrol)}\times 100$$

For the biofilm destruction, a similar experiment was performed but with the incubation of the bacteria in MHB for 24 h and the fungi in PDB for 5 days to form the biofilms before treatment. The antibiofilm activity was also determined using the same mentioned protocol. All experiments were repeated at least three times [19].

### 2.6. Preparation of the Film

An envelope was prepared to preserve the table grapes. Distilled water was boiled and then cooled to room temperature. Following that, 30 g/L of starch and 10% glycerol were added. The solution was heated until the starch gelatinized, and it was allowed to sit until it cooled to room temperature. Finally, the aqueous extract of *C. winterianus* (5 mg/mL and 10 mg/mL) was added to the prepared solution. The grape bunches were washed with sterile distilled water and immersed in the prepared solutions for 2 min. The samples were stored at 4 °C after the package was dried. The results of preservation of the grapes with the extract were monitored after 1, 17, and 37 days of incubation [23].

### 2.7. Statistical Analysis

The statistical analysis and graphs were carried out and drawn, respectively, in Excel (Microsoft office 2016, McIntosh, WA, USA). The statistical significance was determined by *t*-tests. A *p*-value < 0.05 was considered statistically significant.

## 3. Results

### 3.1. Minimum Inhibitory Concentration (MIC) and Minimum Bactericidal Concentration/Minimum Fungicidal Concentration (MBC/MFC) of the Aqueous and Ethanolic O. syriacum and C. winterianus Extracts and EOs

**Figure 1:**
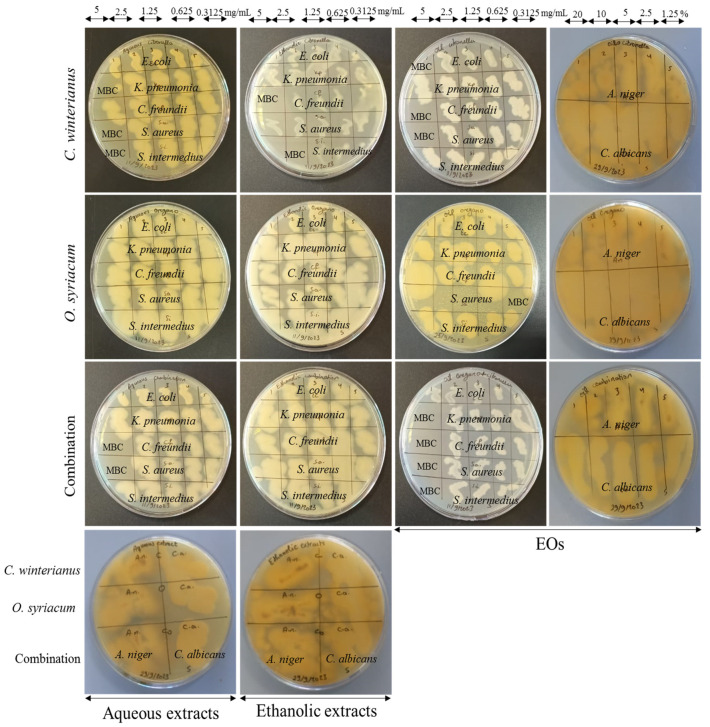
MBC/MFC results of the aqueous and ethanolic *O. syriacum* and *C. winterianus* extracts, and their EOs against the bacterial and fungal isolates (MBC: minimum bactericidal concentration, EOs: essential oils).

The MIC and MBC microdilution assay was performed to detect the bacteriostatic and bactericidal activities of the aqueous and ethanolic *O. syriacum* and *C. winterianus* extracts and their EOs. The aqueous *C. winterianus* extract exhibited a bactericidal effect against *K. pneumonia*, *S. aureus*, and *S. intermedius* (MBCs ranging between 2.5 and 5 mg/mL) and a bacteriostatic effect against *E. coli* and *C. freundii* (MICs ranging between 2.5 and 5 mg/mL). The aqueous *O. syriacum* extract had a bacteriostatic effect against all bacterial isolates (MICs ranging between 1.25 and 2.5 mg/mL). The ethanolic *C. winterianus* extract exhibited a bactericidal effect against *C. freundii* and *S. intermedius* (MBCs ranging between 2.5 and 5 mg/mL) and a bacteriostatic effect against *E. coli*, *K. pneumonia*, and *S. aureus* (MICs ranging between 0.625 and 2.5 mg/mL). Both extracts were able to inhibit bacterial growth at relatively low concentrations. However, better antibacterial activity was recorded for the combined extracts. The aqueous combined extracts exerted bactericidal activity against *C. freundii* and *S. aureus* (MBCs = 5 mg/mL) and a bacteriostatic effect against *E. coli*, *K. pneumonia*, and *S. intermedius* (MICs = 2.5 mg/mL). The ethanolic combined extract showed bacteriostatic activity against all bacterial isolates (MIC ranging between 1.25 and 5 mg/mL). Regarding the EOs of *O. syriacum* and *C. winterianus*, they showed significant inhibitory action against the bacterial isolates, in which they showed bactericidal potentials against most of the isolates at relatively low concentrations. The *O. syriacum* EO had a bactericidal effect against *C. freundii* and *S. aureus* (MBCs ranging between 1.25 and 20%) and bacteriostatic activity against *E. coli*, *K. pneumonia*, and *S. intermedius* (MICs ranging between 1.25 and 2.5 mg/mL). The *C. winterianus* EO had a bactericidal effect against *E. coli*, *C. freundii*, and *S. aureus* (MBCs = 20 mg/mL), and a bacteriostatic effect against *K. pneumonia* and *S. intermedius* (MICs = 2.5 mg/mL). In addition, the combined EOs exerted stronger bactericidal action against all tested isolates (MBCs ranging between 5 and 20%), except *E. coli*. For the fungal isolates, *C. albicans* was more resistant than *A. niger*. Both aqueous and ethanolic extracts had fungistatic activity with a better effect for the ethanolic extracts. The aqueous *O. syriacum*, *C. winterianus*, and combined extracts showed inhibitory action against *A. niger* only (MIC = 5 mg/mL). However, the ethanolic extracts had a fungistatic effect against both *C. albicans* and *A. niger* (MICs ranging between 2.5 and 5 mg/mL). On the other hand, the EOs had significant inhibitory activity with fungistatic action against *C. albicans* (MICs ranging between 1.25 and 5%) and fungicidal activity against *A. niger* (MBCs = 10%). The MIC and MBC/MFC results are presented in Table 1 and Figure 1, Figures S1 and S2. Table 2 shows a comparison between the MICs of the extracts and EOs used in this study and the MICs of similar extracts and EOs used on the same bacterial and fungal species from previous studies. In addition, all data were significant with *p*-values < 0.05, as presented in Table S1.

### 3.2. Time-Kill Results of the Aqueous and Ethanolic O. syriacum and C. winterianus Extracts and EOs against the Bacterial and Fungal Isolates

The time-kill assay was performed to detect the time needed by the aqueous and ethanolic *O. syriacum* and *C. winterianus* extracts and their EOs to exert their bacteriostatic and bactericidal/fungicidal actions. In general, both aqueous and ethanolic extracts needed at least 1 h to inhibit bacterial growth. Gram-positive bacteria were inhibited faster than Gram-negative bacteria. The aqueous extracts inhibited bacterial growth within 2–4 h, while the ethanolic extracts took 24 h to exert their inhibitory action. Similar to the aqueous extracts and in contrast to the ethanolic extracts, the EOs inhibited bacterial growth within only 1 h, with a better effect for the combined samples. For the fungal isolates, the mean time needed for inhibition ranged between 1 and 4 h. The ethanolic extracts were faster in inhibiting the growth of fungi than the aqueous extracts. However, the combined extracts took more time to inhibit *C. albicans* and *A. niger*. The results of the time-kill assay against the bacterial and fungal isolates are presented in Table 3 and Figures S3 and S4. In addition, the significant *p*-values are presented in Table S2.

### 3.3. Antibiofilm Activity of the Aqueous and Ethanolic O. syriacum and C. winterianus Extracts and EOs against the Bacterial and Fungal Isolates

**Figure 2:**
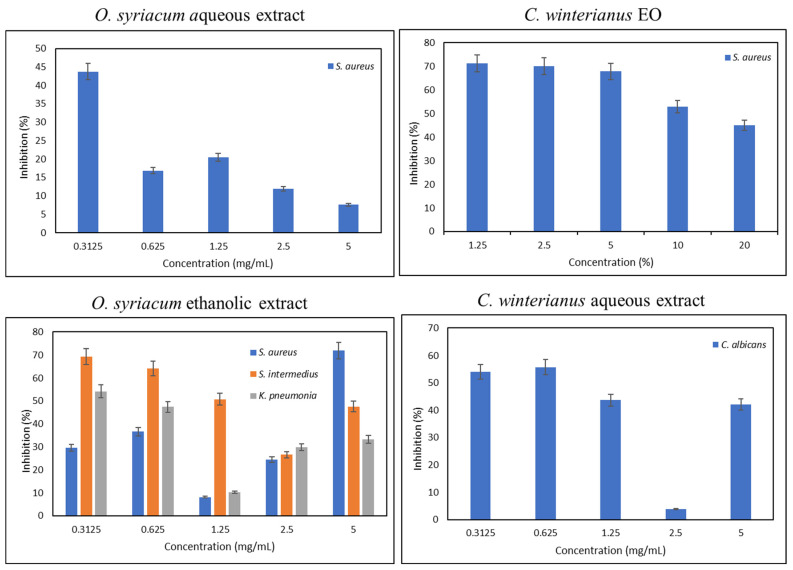
Inhibition of the formation of bacterial and fungal biofilms by the aqueous and ethanolic *O. syriacum* and *C. winterianus* extracts and their EOs (EO: essential oil).

The antibiofilm assays were performed to determine the ability of the aqueous and ethanolic *O. syriacum* and *C. winterianus* extracts and their EOs to inhibit and destroy biofilms. In contrast to bacterial isolates, biofilms showed great resistance to both *O. syriacum* and *C. winterianus* extracts, as well as their EOs. Among the aqueous extracts, only *O. syriacum* was able to inhibit the formation of *S. aureus* biofilm at a concentration of 0.3125 mg/mL. Similarly, among the ethanolic extracts, *O. syriacum* was able to inhibit the formation of *S. intermedius* biofilm at a concentration of 0.3125 mg/mL and *K. pneumonia* biofilm at a concentration of 0.3125 mg/mL. Unfortunately, among the combined extracts, the ethanolic extract was only able to inhibit the formation of *S. aureus* biofilm at a concentration of 5 mg/mL. As for the fungal biofilms, the *C. winterianus* aqueous extract was only able to inhibit the formation of *C. albicans* biofilm at a concentration of 0.625 mg/mL. However, the *A. niger* biofilm was resistant to all extracts and EOs. The other extracts and EOs did not show any inhibitory activity. The inhibitory percentages are presented in Figure 2, and the significant *p*-values of the antibiofilm formation are presented in Table S3.

### 3.4. Destruction of Pre-Formed Biofilms

**Figure 3:**
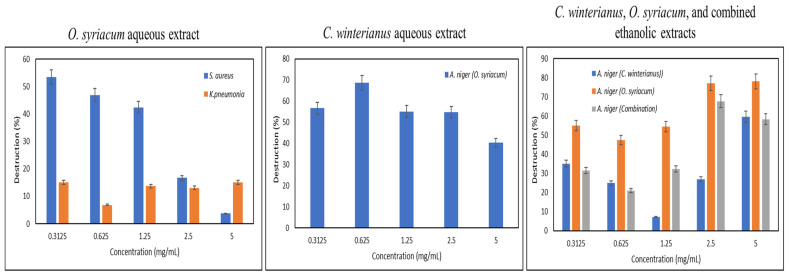
Destruction of the pre-formed bacterial and fungal biofilms by the aqueous and ethanolic *O. syriacum* and *C. winterianus* extracts and their EOs (EO: essential oil).

Similar to the inhibition of biofilm formation, the aqueous and ethanolic *O. syriacum* and *C. winterianus* extracts showed weak destructive activity against almost all the bacterial biofilms. However, the *O. syriacum* aqueous extract was able to destroy the *S. aureus* biofilm at a concentration of 0.3125 mg/mL and the *K. pneumonia* biofilm at a concentration of 0.3125 mg/mL. As for the pre-formed fungal biofilms, the aqueous *C. winterianus* extract was able to destroy the *C. albicans* biofilm only at a concentration of 0.625 mg/mL. However, *A. niger* biofilm showed sensitivity to the combined aqueous extract at a concentration of 0.625 mg/mL, as well as to all ethanolic extracts at concentrations ranging between 2.5 and 5 mg/mL. The destruction percentages are presented Figure 3, and the significant *p*-values of the antibiofilm formation are presented in Table S4.

### 3.5. Preservation of the Table Grapes by the Prepared Film

**Figure 4:**
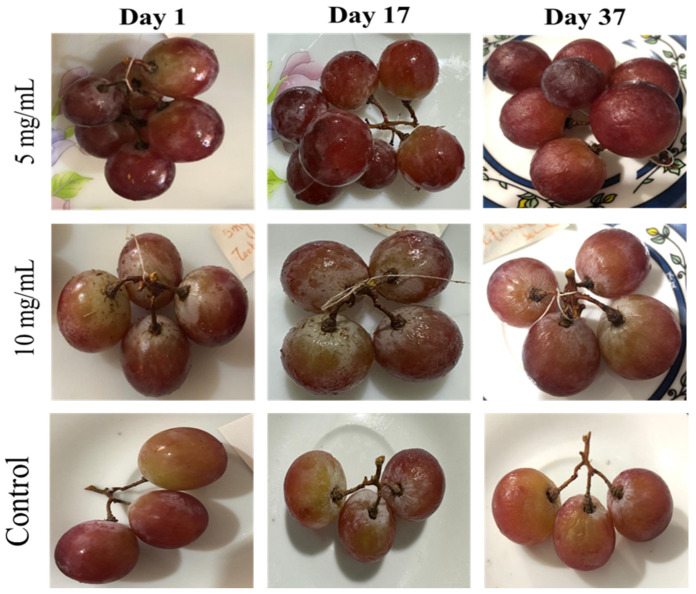
Appearance of the grapes’ structures after packaging with the aqueous *C. winterianus* extracts.

The appearance of grape berries was improved in terms of brightness and color at the beginning of the shelf life (1st day). The best external appearance of the grape bunches after having been stored for 37 days in the refrigerator at 4 °C was in the bunches wrapped in the packaging prepared with the aqueous extract of *C. winterianus* at a concentration of 10 mg/mL. This gives the grape clusters the ability to maintain skin consistency compared to the rest of the clusters, where the berries lose their firmness and become wrinkled. The preservation results are presented in Figure 4.

## 4. Discussion

In the framework of studying the antibacterial and antifungal activity of *C. winterianus* and *O. syriacum* extracts and their EOs, they were tested against five bacterial isolates (*E. coli*, *K. pneumonia*, *C. freundii*, *S. aureus*, and *S. intermedius*) isolated from urine samples and two fungal isolates (*C. albicans* and *A. niger*) isolated from spoiled food samples. The mentioned extracts and their EOs proved to be effective antimicrobial agents against bacteria and fungi [19]. However, the antimicrobial activity of the combined *C. winterianus* and *O. syriacum* extracts and EOs has not been taken into consideration previously. So, this study aimed to check the antimicrobial efficacy of the combined *C. winterianus* and *O. syriacum* extracts, as well as their EOs. *C. winterianus* and *O. syriacum* extracts and their EOs, like other natural extracts and oils, were proved to be not toxic, safe, and friendly to the environment [19,29,30]. This study revealed that *C. winterianus* and *O. syriacum* extracts and their EOs, especially their combinations, exhibited significant antibacterial and antifungal activities. The aqueous and ethanolic *C. winterianus* and *O. syriacum* extracts exhibited bacteriostatic activity, while the EOs had mostly bactericidal action against the tested isolates. This antimicrobial efficacy is caused by the presence of phenolic and monoterpene components in the extracts and the EOs, especially carvacrol and thymol [13,30]. Carvacrol was proved to be the most effective component against microbes. Thymol and carvacrol were proved to exert toxic effects, especially against *E. coli* and *S. aureus*, with a stronger action by carvacrol [14].

In addition, these bactericidal and bacteriostatic activities could be due to the hydrophobic nature of the extracts and EOs, which facilitate their penetration into the bacterial cell, thus changing the orientation of lipids in the cell membrane [14]. These changes alter the chemical and physical properties of the cell membrane, which increases the electron flow and proton flux across the membrane, thus coagulating the cell contents and causing cell death [14,19]. Among the tested bacterial isolates, *S. aureus* was the most sensitive bacterium and *E. coli* was the most resistant. This means that *C. winterianus* and *O. syriacum* extracts and EOs exerted better inhibitory activity against Gram-positive bacteria than Gram-negative bacteria. Gram-negative bacteria are more resistant than Gram-positive bacteria due to the presence of an outer membrane rich in lipopolysaccharides in the Gram-negative bacterial cell wall [19,31,32].

**Figure 5:**
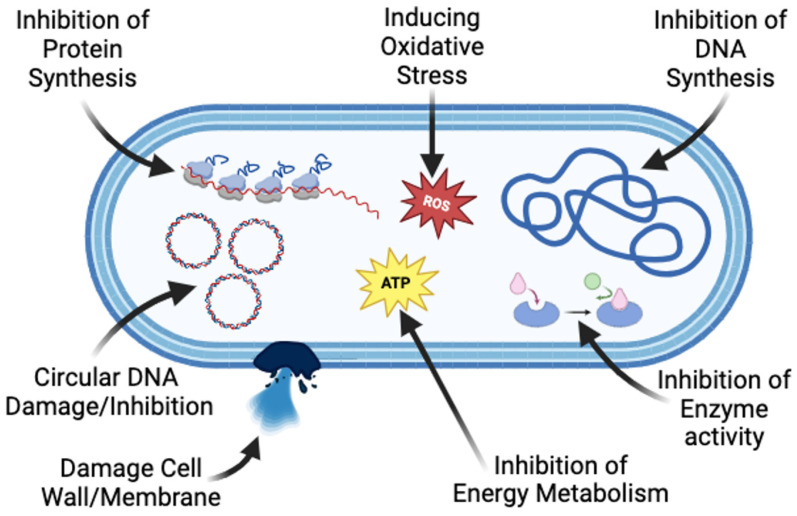
Proposed mechanisms of action of extracts against bacteria (created with BioRender.com, accessed on 16 May 2024) modified from Liang et al. [38].

For fungal susceptibility, *O. syriacum* extract and its EO were proved to show efficacy against many fungal isolates [16]. The mechanism of action is similar to that of bacteria. For example, Daouk et al. reported that the presence of thymol and carvacrol in *O. syriacum* extracts plays a vital role in the antimicrobial activity of the extract as well as the EO, especially against *Penicillium* and *Fusarium* [16]. The cytotoxic activity was attributed to their lipophilic nature, which enables them to penetrate the cell membrane of fungi leading to damage of the cellular components. In addition, the penetration of the EOs into the cell leads to interaction with cellular ions, leading to depletion of the ATP pool and the leakage of calcium, magnesium, and potassium ions, thus damaging the cells and causing cell lysis [16,19]. Another study reported the effective antifungal activity of *O. syriacum* and many species of *Citronella* EOs against *A. niger*. They inhibited the mycelial growth of fungi [16,17]. This effect was specifically shown to be due to the presence of carvacrol and thymol, in addition to many other compounds including sesquiterprenoids, monoterpenoids, eudesmol, geraniol, and geranyl acetate [17]. The mentioned compounds were proved to work in a cascade of reactions. Dangol et al. reported some antagonistic mechanisms including the disruption of the cytoplasmic membrane leading to leakage and the inhibition of sporulation, thus leading to cell lysis [17]. Other mechanisms include changes in the permeability and damaging the cell membrane, inhibition of the respiratory metabolism, forming chimeras with the DNA (especially carvacrol), degenerating the cell morphology, and decreasing the total protein amount [33,34]. The proposed mechanisms of the antimicrobial actions are illustrated in Figure 5.

It is worth mentioning that the action of the extracts, as well as their EOs, depends on their chemical composition. As mentioned previously, *O. syriacum* and *C. winterianus* are rich in phenolic compounds. Previous literature reported that carvacrol, thymol, cymene, terpinene, myrcene, caryophyllene, and thymoquinones are the main constituents of *O. syriacum* and *C. winterianus* extracts [26,33,34]. For example, Mesmar et al. [15] listed the mentioned chemical compounds and reported that they are responsible for the antibacterial and antifungal mechanisms exerted by the extracts.

In addition, the chemical composition of the EOs plays a vital role in their antibacterial and antifungal activities. Rammal et al. showed in a recent study that the main chemical compounds revealed in the *O. syriacum* and *C. winterianus* EOs include geraniol and citronellol, along with many other compounds found in lower percentages. The compounds are responsible for the antibacterial and antifungal mechanisms exerted by the *O. syriacum* and *C. winterianus* EOs.

The time-kill results revealed that all isolates required 1 to 4 h to be inhibited. Usually, bacteria need at least 12 h for maturation and fungi need about 48 h for full growth. However, both bacteria and fungi are able to fix to their surface within 4 h. This time is needed for nutrient uptake. This means that the extracts and their oils inhibited the nutrient uptake by bacteria and fungi, thus inhibiting their attachment and growth [21,22].

Regarding the antibiofilm results, unfortunately, the *C. winterianus* and *O. syriacum* extracts and their EOs had a very weak inhibitory activity against almost all biofilms. They neither inhibited the biofilm formation nor destroyed the pre-formed biofilms. However, the exerted inhibitory activity against some biofilms is explained by the fact that natural extracts and EOs are able to interact with the exopolysaccharides secreted by biofilms to prevent their attachment [19,35,36]. In addition, the inhibitory concentrations recorded were independent of the MICs and MBCs/MFCs. This is attributed to the fact that anti-biofilm activities and concentrations are independent due to the variation in absorbances and cell enumeration [37]. The interaction between the major components of the extracts and EOs leads to inhibitory activities. However, the weak activity could be explained by the fact that biofilms create more resistance due to the formation of clusters of cells making the bacteria and fungi stronger [35,36,37].

Grapes are non-climacteric perishable fresh food products with several other post-harvest storage problems such as loss of firmness, berry dropping, stem discoloration, and desiccation. *C. winterianus* extract was able to protect the skin of the berries more than *O. syriacum* extract. We can consider this film as a concentration of polyphenols including flavones, anthocyanins (both coloring pigments), and stilbenes such as resveratrol. In nature, polyphenols have essential missions for survival. In addition to their defense against environmental attacks (ultraviolet radiation, invasion by insects, fungi, and viruses), polyphenols also perform “appetizing” functions. They are partly responsible for the sensory and nutritional qualities of plants including astringency, color, and odor. In the flavonoid family, anthocyanins are responsible for the red color of grapes; resveratrol, in its natural combination in whole grapes, has antifungal properties. It may be that the *C. winterianus* extract was able to preserve them.

Overall, the *C. winterianus* and *O. syriacum* extracts and their EOs had significant antibacterial and antifungal activities. These effects allow for their use in food preservation and protection against bacterial and fungal spoilage.

## 5. Conclusions

This study reported the antibacterial and antifungal potentials of *C. winterianus* and *O. syriacum* extracts and their EOs against various uropathogenic bacteria and foodborne fungi. In addition, their preservative strength was tested on grapes. For this, their antibacterial activity was detected against five bacteria isolated from urine (*E. coli*, *K. pneumonia*, *C. freundii*, *S. aureus*, and *S. intermedius*). Both the aqueous and ethanolic *O. syriacum* and *C. winterianus* extracts exhibited bacteriostatic and bactericidal actions against the tested isolates. Furthermore, the EOs showed better inhibitory activity than the extracts on the same isolates. Their action was exerted within 2 to 4 h of incubation with the bacteria. However, the extracts and EOs exerted weak antibiofilm activity against the bacterial biofilms. They were neither able to inhibit the formation of the biofilms nor eradicate the pre-formed biofilms. For the antifungal activities, two foodborne fungi isolated from lemon and jam were tested (*A. niger* and *C. albicans*). The *O. syriacum* and *C. winterianus* extracts and EOs exerted fungistatic actions against the tested fungi. However, the EOs exerted a stronger action than the extracts. Their action was reported after 3 to 4 h of incubation. Their antibiofilm action against the fungal isolates was relatively weak. For the packaging potential, the *O. syriacum* and *C. winterianus* extracts were able to preserve the grapes for about 40 days, with a better effect for *C. winterianus*. It is worth mentioning that the combined samples of the two extracts and their EOs had better potential against bacteria and fungi than the individual *O. syriacum* and *C. winterianus*. This study opens an avenue for the use of combined *O. syriacum* and *C. winterianus* extracts and EOs against different uropathogenic and foodborne microorganisms and their use in food preservation.

## Figures and Tables

**Table 1:** MICs and MBCS/MFCs of the aqueous and ethanolic *O. syriacum* and *C. winterianus* extracts (mg/mL) and their EOs (%) against the bacterial and fungal isolates.

Plant Extracts and EOs	MICs and MBCs/MFCs of the Extracts (mg/mL) and the Oils (%)	Bacterial Isolates					Fungal Isolates	
		Gram-Negative Bacteria			Gram-Positive Bacteria			
		*E. coli*	*K. pneumonia*	*C. freundii*	*S. aureus*	*S. intermedius*	*C. albicans*	*A. niger*
*C. winterianus* AE	MIC	5	2.5	2.5	2.5	2.5	-	5
	MBC/MFC	-	5	-	5	5	-	-
*O. syriacum* AE	MIC	2.5	2.5	1.25	1.25	1.25	-	5
	MBC/MFC	-	-	-	-	-	-	-
Combination of AE	MIC	2.5	2.5	1.25	2.5	2.5	-	5
	MBC/MFC	-	-	5	5	-	-	-
*C. winterianus* EE	MIC	2.5	1.25	2.5	0.625	1.25	5	5
	MBC/MFC	-	-	5	-	2.5	-	-
*O. syriacum* EE	MIC	-	2.5	1.25	1.25	2.5	5	5
	MBC/MFC	-	-	-	-	-	-	-
Combination of EE	MIC	2.5	1.25	2.5	5	5	2.5	5
	MBC/MFC	-	-	-	-	-	-	-
*C. winterianus* EO	MIC	2.5	2.5	5	2.5	2.5	1.25	1.25
	MBC/MFC	20	-	20	20	-	-	10
*O. syriacum* EO	MIC	2.5	1.25	1.25	-	1.25	2.5	5
	MBC/MFC	-	-	20	1.25	-	-	10
Combination of EOs	MIC	20	5	5	5	5	5	5
	MBC/MFC	-	20	20	20	20	-	10

EOs: essential oils, AE: Aqueous extract, EE: ethanolic extract, MIC: minimum inhibitory concentration, MBC: minimum bactericidal concentration, MFC: minimum fungicidal concentration, “-”: not determined.

**Table 2:** Comparison between the MICs of some extracts and EOs of the same origin as *O. syriacum* and *C. winterianus* from previous literature.

Plant	Extracts and EOs	MICs of Extracts and EOs					Reference
		Bacterial Isolates			Fungal Isolates		
		*E. coli*	*K. pneumonia*	*S. aureus*	*A. niger*	*C. albicans*	
*Origanum*	EO	0.5 mg/mL	0.5 mg/mL	0.5 mg/mL	-	-	[24]
*Monolaurin*	EO	>8 mg/mL	>8 mg/mL	0.063 mg/mL	-	-	
*O. syriacum*	AE	12.5 µg/mL	6.25 C	-	-	-	[25]
*O. syriacum*	EO	-	40 µg/mL	-	-	-	[26]
*C. khasianus*	EO	20 V	20 µg/mL	30 µg/mL	-	100 µg/mL	[27]
*C. winterianus*	EO	0.98 µg/mL	-	0.98 µg/mL	-	-	[28]
*C. winterianus*	EO	6 µg/mL	-	2 µg/mL	2.5 µg/mL	1 µg/mL	[29]
*O. syriacum* and *C. winterianus*	Combined AEs	2.5 mg/mL	2.5 mg/mL	2.5 mg/mL	5 mg/mL	-	Current study
	Combined EEs	2.5 mg/mL	1.25 mg/mL	5 mg/mL	5 mg/mL	2.5 mg/mL	
	Combined EOs	20%	5%	5%	5%	5%	

MIC: minimum inhibitory concentration, EOs: essential oils, AE: Aqueous extract, EE: ethanolic extract, “-”: not determined.

**Table 3:** Time-kill results of the aqueous and ethanolic *O. syriacum* and *C. winterianus* extracts and their EOs against the bacterial and fungal isolates.

Plant Extracts and Oils	Time of Inhibition (h) of Bacterial and Fungal Isolates						
	Bacterial Isolates					Fungal Isolates	
	Gram-Negative Bacteria			Gram-Positive Bacteria			
	*E. coli*	*K. pneumonia*	*C. freundii*	*S. aureus*	*S. intermedius*	*C. albicans*	*A. niger*
*C. winterianus* AE	24	2	24	2	2	4	2
*O. syriacum* AE	24	1	2	2	2	1	1
Combination of AEs	1	1	1	2	24	2	1
*C. winterianus* EE	24	24	24	24	24	1	1
*O. syriacum* EE	24	24	24	24	24	2	1
Combination of EEs	24	24	24	24	24	2	2
*C. winterianus* EO	2	2	4	1	1	1	1
*O. syriacum* EO	1	2	4	24	2	2	2
Combination of EOs	1	4	1	1	2	4	4

EOs: essential oils, AE: Aqueous extract, EE: ethanolic extract.

## Data Availability

The original contributions presented in the study are included in the article/Supplementary Materials, further inquiries can be directed to the corresponding authors.

## Supplementary Materials

The following supporting information can be downloaded at: https://www.mdpi.com/article/10.3390/foods13111684/s1.

## References

[1] Gontard N., Guillard V., Gaucel S., Guillaume C. (2017). L’emballage alimentaire et l’innovation écologique dans toutes leurs dimensions. Innov. Agron..

[2] Odjo K., Al-Maqtari Q.A., Yu H., Xie Y., Guo Y., Li M., Du Y., Liu K., Chen Y., Yao W. (2022). Preparation and characterization of chitosan-based antimicrobial films containing encapsulated lemon essential oil by ionic gelation and cranberry juice. Food Chem..

[3] Perera K.Y., Jaiswal A.K., Jaiswal S. (2023). Biopolymer-Based Sustainable Food Packaging Materials: Challenges, Solutions, and Applications. Foods.

[4] Avramescu S.M., Butean C., Popa C.V., Ortan A., Moraru I., Temocico G. (2020). Edible and functionalized films/coatings-performances and perspectives. Coatings.

[5] Chaudhary P., Fatima F., Kumar A. (2020). Relevance of Nanomaterials in Food Packaging and Its Advanced Future Prospects. J. Inorg. Organomet. Polym. Mater..

[6] Hou X., Xue Z., Xia Y., Qin Y., Zhang G., Liu H., Li K. (2019). Effect of SiO_2_ nanoparticle on the physical and chemical properties of eco-friendly agar/sodium alginate nanocomposite film. Int. J. Biol. Macromol..

[7] Mostafavi F.S., Zaeim D. (2020). Agar-based edible films for food packaging applications—A review. Int. J. Biol. Macromol..

[8] Breceda-Hernandez T.G., Martínez-Ruiz N.R., Serna-Guerra L., Hernández-Carrillo J.G. (2020). Effect of a pectin edible coating obtained from orange peels with lemon essential oil on the shelf life of table grapes (*Vitis vinifera* L. var. Red. Globe). Int. Food Res. J..

[9] Arnon H., Granit R., Porat R., Poverenov E. (2015). Development of polysaccharides-based edible coatings for citrus fruits: A layer-by-layer approach. Food Chem..

[10] Nottagh S., Hesari J., Peighambardoust S.H., Rezaei-Mokarram R., Jafarizadeh-Malmiri H. (2020). Effectiveness of edible coating based on chitosan and Natamycin on biological, physico-chemical and organoleptic attributes of Iranian ultra-filtrated cheese. Biologia.

[11] Karaca H., Pérez-Gago M.B., Taberner V., Palou L. (2014). Evaluating food additives as antifungal agents against Monilinia fructicola in vitro and in hydroxypropyl methylcellulose-lipid composite edible coatings for plums. Int. J. Food Microbiol..

[12] do Nascimento A., Toneto L.C., Lepaus B.M., Valiati B.S., Faria-Silva L., de São José J.F.B. (2023). Effect of Edible Coatings of Cassava Starch Incorporated with Clove and Cinnamon Essential Oils on the Shelf Life of Papaya. Membranes.

[13] Yaradoddi J., Banapurmath N., Ganachari D.S., Soudagar M.E., Sajjan A., Kamat S., Abbas M.M., Shettar A., Anqi A., Safaei M.R. (2021). Bio-based Material from Fruit Waste of Orange Peel for Industrial Applications. J. Mater. Res. Technol..

[14] Mohamad R., Mussa R., Suslina S.N. (2021). Prospects for using *Origanum syriacum* (L.) as a source of antimicrobial agents. J. Adv. Pharm. Technol. Res..

[15] Mesmar J., Abdallah R., Badran A., Maresca M., Baydoun E. (2022). *Origanum syriacum* Phytochemistry and Pharmacological Properties: A Comprehensive Review. Molecules.

[16] Daouk R.K., Dagher S.M., Sattout E.J. (1995). Antifungal Activity of the Essential Oil of *Origanum syriacum* L.. J. Food Prot..

[17] Dangol S., Poudel D.K., Ojha P.K., Maharjan S., Poudel A. (2023). Essential Oil Composition Analysis of Cymbopogon Species from Eastern Nepal by GC-MS and Chiral GC-MS, and Antimicrobial Activity of Some Major Compounds. Molecules.

[18] Liu D., Zhao P., Chen J., Yan Y., Wu Z. (2022). Recent Advances and Applications in Starch for Intelligent Active Food Packaging: A Review. Foods.

[19] Mezher M., El Hajj R., Khalil M. (2022). Investigating the antimicrobial activity of essential oils against pathogens isolated from sewage sludge of southern Lebanese villages. Germs.

[20] Rammal M., Badran A., Haidar C., Sabbah A., Bechelany M., Awada M., Hassan K.H., El-Dakdouki M., Raad M.T. (2024). *Cymbopogon winterianus* (Java Citronella Plant): A Multi-Faceted Approach for Food Preservation, Insecticidal Effects, and Bread Application. Foods.

[21] Adnan R.M., Mezher M., Abdallah A.M., Awad R., Khalil M.I. (2023). Synthesis, Characterization, and Antibacterial Activity of Mg-Doped CuO Nanoparticles. Molecules.

[22] Adnan R., Abdallah A.M., Mezher M., Noun M., Khalil M., Awad R. (2023). Impact of Mg-doping on the structural, optical, and magnetic properties of CuO nanoparticles and their antibiofilm activity. Phys. Scr..

[23] Tarique J., Sapuan S.M., Khalina A. (2021). Effect of glycerol plasticizer loading on the physical, mechanical, thermal, and barrier properties of arrowroot (*Maranta arundinacea*) starch biopolymers. Sci. Rep..

[24] Preuss H.G., Echard B., Enig M., Brook I., Elliott T.B. (2005). Minimum inhibitory concentrations of herbal essential oils and monolaurin for gram-positive and gram-negative bacteria. Mol. Cell. Biochem..

[25] Al-Mariri A., Safi M. (2014). In Vitro Antibacterial Activity of Several Plant Extracts and Oils against Some Gram-Negative Bacteria. Iran. J. Med. Sci..

[26] AL-Mariri A., Odeh A., Alobeid B., Boukai H. (2019). In Vitro Antibacterial Activity of *Origanum syriacum* L. Essential Oils against Gram-Negative Bacteria. Avicenna J. Clin. Microbiol. Infect..

[27] Singh G., Katoch M. (2020). Antimicrobial activities and mechanism of action of *Cymbopogon khasianus* (Munro ex Hackel) Bor essential oil. BMC Complement. Med. Ther..

[28] Mangalagiri N.P., Panditi S.K., Jeevigunta N.L.L. (2021). Antimicrobial activity of essential plant oils and their major components. Heliyon.

[29] Simic A., Rančic A., Sokovic M.D., Ristic M., Grujic-Jovanovic S., Vukojevic J., Marin P.D. (2008). Essential Oil Composition of *Cymbopogon winterianus* and *Carum carvi* and Their Antimicrobial Activities. Pharm. Biol..

[30] Swamy M.K., Akhtar M.S., Sinniah U.R. (2016). Antimicrobial properties of plant essential oils against human pathogens and their mode of action: An updated review. Evidence-based Complement. Altern. Med..

[31] Bhavaniramya S., Vishnupriya S., Al-Aboody M.S., Vijayakumar R., Baskaran D. (2019). Role of essential oils in food safety: Antimicrobial and antioxidant applications. Grain Oil Sci. Technol..

[32] Nazzaro F., Fratianni F., De Martino L., Coppola R., De Feo V. (2013). Effect of essential oils on pathogenic bacteria. Pharmaceuticals.

[33] Anastasiou T.I., Mandalakis M., Krigas N., Vézignol T., Lazari D., Katharios P., Dailianis T., Antonopoulou E. (2019). Comparative Evaluation of Essential Oils from Medicinal-Aromatic Plants of Greece: Chemical Composition, Antioxidant Capacity and Antimicrobial Activity against Bacterial Fish Pathogens. Molecules.

[34] Coccimiglio J., Alipour M., Jiang Z.-H., Gottardo C., Suntres Z. (2016). Antioxidant, Antibacterial, and Cytotoxic Activities of the Ethanolic *Origanum vulgare* Extract and Its Major Constituents. Oxidative Med. Cell. Longev..

[35] Iseppi R., Tardugno R., Brighenti V., Benvenuti S., Sabia C., Pellati F., Messi P. (2020). Phytochemical composition and in vitro antimicrobial activity of essential oils from the lamiaceae family against streptococcus agalactiae and candida albicans biofilms. Antibiotics.

[36] Famuyide I.M., Aro A.O., Fasina F.O., Eloff J.N., McGaw L.J. (2019). Antibacterial and antibiofilm activity of acetone leaf extracts of nine under-investigated south African *Eugenia* and *Syzygium* (Myrtaceae) species and their selectivity indices. BMC Complement. Altern. Med..

[37] Aires A., Barreto A.S., Semedo-Lemsaddek T. (2021). Antimicrobial effects of essential oils on oral microbiota biofilms: The toothbrush in vitro model. Antibiotics.

[38] Liang J., Huang X., Ma G. (2022). Antimicrobial activities and mechanisms of extract and components of herbs in East Asia. RSC Adv..

