# Supplementary material for: Antibacterial and Antifungal Activities of Cimbopogon winterianus and Origanum syriacum Extracts and Essential Oils against Uropathogenic Bacteria and Foodborne Fungal Isolates
Source: Foods. 2024 May 27;13(11):1684. doi: 10.3390/foods13111684 (PMC11171924; doi:10.3390/foods13111684)
Supplement: Supplementary file 1 [file foods-13-01684-s001.zip › foods-2998124-supplementary.pdf]

Supplementary Information

Antibacterial and Antifungal Activities of *Cimbopogon winterianus* and *Origanum syriacum* Extracts and Essential Oils against Uropathogenic Bacteria and Foodborne Fungal Isolates

Marwa Rammal [1], Salam Khreiss [1], Adnan Badran [2], Malak Mezher [3], Mikhael Bechelany [4,5,*], Chaden Haidar [1], Mahmoud I. Khalil [3,6], Elias Baydoun [7] and Mohammad H. El-Dakdouki [8,*]

Table S1. *p*-values and significance levels of the MICs of the aqueous and ethanolic *O. syriacum* and *C. winterianus* extracts and their EOs.

Extracts and EOs		Bacterial isolates										Fungal isolates			
		E. coli	S	*K. pneumonia*	S	*C. freundii*	S	*S. aureus*	S	*S. intermedius*	S	*C. albicans*	S	*A. niger*	S
Sample	Concentration (mg/mL for extracts and % for EOs)	*p*-value (vs. water for the aqueous extracts, ethanol for the ethanolic extracts, and DMSO for the EOs)													
O. syriacum aqueous extract	5	0.01	**	0.001	***	0.001	***	0.001	***	0.05	*	0.001	***	0.01	**
	2.5	0.003	***	0.002	**	0.01	**	0.02	*	0.05	*	0.02	*	0.01	**
	1.25	0.02	*	0.001	***	0.04	*	0.01	**	0.02	*	0.01	**	0.001	***
	0.625	0.05	*	0.001	***	0.02	*	0.003	**	0.001	***	0.02	*	0.05	*
	0.3125	0.002	**	0.001	***	0.001	***	0.006	**	0.001	***	0.002	**	0.02	*
C. winterianus aqueous extract	5	0.02	*	0.01	**	0.001	***	0.001	***	0.005	**	0.005	**	0.006	**
	2.5	0.05	*	0.05	*	0.03	**	0.005	**	0.05	*	0.005	**	0.002	**
	1.25	0.001	***	0.02	*	0.001	***	0.01	**	0.03	*	0.001	***	0.006	**
	0.625	0.01	**	0.002	**	0.02	*	0.02	*	0.02	*	0.001	***	0.005	**
	0.3125	0.002	**	0.03	*	0.01	**	0.04	*	0.02	*	0.05	*	0.001	***
Combination of aqueous extracts	5	0.02	*	0.04	*	0.02	*	0.05	*	0.001	***	0.01	**	0.001	***
	2.5	0.002	**	0.001	***	0.001	***	0.05	*	0.001	***	0.01	**	0.02	*
	1.25	0.004	**	0.001	***	0.002	**	0.05	*	0.001	***	0.006	**	0.02	*
	0.625	0.05	*	0.001	***	0.003	**	0.001	***	0.02	*	0.05	*	0.02	*
	0.3125	0.001	***	0.02	**	0.01	**	0.001	***	0.002	**	0.02	*	0.001	***
O. syriacum ethanolic extract	5	0.01	**	0.01	**	0.001	***	0.005	**	0.002	**	0.03	*	0.001	***
	2.5	0.001	***	0.001	***	0.02	*	0.006	**	0.05	*	0.001	***	0.004	**
	1.25	0.05	*	0.001	***	0.02	*	0.005	**	0.006	**	0.002	**	0.001	***
	0.625	0.03	*	< 0.001	***	0.001	***	0.02	*	0.003	**	0.002	**	0.001	***
	0.3125	0.01	**	< 0.001	***	0.005	**	0.02	*	0.01	**	0.005	**	0.02	*
	5	0.001	***	0.01	**	0.005	**	0.03	*	0.01	**	0.004	**	0.03	*

C. winterianus ethanolic extract	2.5	0.001	***	0.02	**	0.001	***	0.001	***	0.02	*	0.01	**	0.03	*
	1.25	0.02	*	0.05	*	0.006	**	0.001	***	0.001	***	0.02	*	0.006	**
	0.625	0.003	**	0.02	*	0.01	**	0.02	*	0.001	***	0.03	*	0.005	**
	0.3125	0.05	*	0.001	***	0.006	**	0.002	**	0.001	***	0.01	**	0.001	***
Combination of ethanolic extracts	5	0.02	*	0.001	***	0.01	**	0.03	*	0.001	***	0.001	***	0.001	***
	2.5	0.001	***	0.001	***	0.02	*	0.03	*	0.02	*	0.03	*	0.02	*
	1.25	0.001	***	0.02	*	0.03	*	0.03	*	0.04	*	0.005	**	0.05	*
	0.625	0.03	*	0.05	*	0.001	***	0.001	***	0.03	*	0.006	**	0.04	*
	0.3125	0.05	*	0.05	*	0.004	**	0.001	***	0.03	*	0.001	***	0.05	*
O. syriacum EO	20	0.001	***	0.02	*	0.005	**	0.02	*	0.006	**	0.002	**	0.001	***
	10	0.001	***	0.01	**	0.05	*	0.05	*	0.001	***	0.001	***	0.005	***
	5	0.001	***	0.03	*	0.02	*	0.02	*	0.005	**	0.01	**	0.002	**
	2.5	0.001	***	0.04	*	0.002	**	0.001	***	0.005	**	0.01	**	0.004	**
	1.25	0.02	*	0.01	**	0.001	***	0.05	*	0.005	**	0.01	**	0.001	***
C. winterianus EO	20	0.05	*	0.001	***	0.001	***	0.006	**	0.001	***	0.001	***	0.004	**
	10	0.003	**	0.05	*	0.006	**	0.01	**	0.02	*	0.005	**	0.002	**
	5	0.004	**	0.05	*	0.004	**	0.02	*	0.03	*	0.001	***	0.001	***
	2.5	0.05	*	0.04	*	0.01	**	0.02	*	0.02	*	0.05	*	0.01	**
	1.25	0.01	**	0.01	**	0.05	*	0.001	***	0.006	**	0.02	*	0.01	**
Combination of EOs	20	0.001	***	0.001	***	0.01	**	0.002	**	0.01	**	0.001	***	0.02	*
	10	0.002	**	0.001	***	0.01	**	0.001	***	0.001	***	0.002	**	0.01	**
	5	0.003	**	0.02	*	0.02	*	0.01	**	0.002	**	0.006	**	0.005	**
	2.5	0.01	**	0.02	*	0.02	*	0.001	***	0.001	***	0.006	**	0.005	**
	1.25	0.001	***	0.001	***	0.001	***	0.002	***	0.05	*	0.001	***	0.01	**

MICs: minimum inhibitory concentrations, DMSO: dimethyl sulfoxide, EOs: essential oils, S: significance level, ***: $p < 0.001$, **: $p < 0.01$, *: $p < 0.05$.

Table S2. *p*-values and significance levels of the time of killing of the aqueous and ethanolic *O. syriacum* and *C. winterianus* extracts and their EOs.

Extracts and EOs		Bacterial isolates										Fungal isolates			
		E. coli	S	*K. pneumonia*	S	*C. freundii*	S	*S. aureus*	S	*S. intermedius*	S	*C. albicans*	S	*A. niger*	S
Sample	Time (h)	*p*-value (vs. time)													
O. syriacum aqueous extract	1	-	-	0.006	**	-	-	-	-	-	-	0.002	**	0.001	***
	2	-		-	-	0.004	**	0.001	***	0.002	**	-	-	-	-
	24	0.02	*	-	-	-	-	-	-	-	-	0.002	**	0.02	*
C. winterianus aqueous extract	2	-	-	0.002	**	-	-	0.004	**	0.003	**	-	-	0.05	*
	4	-	-	-		-	-	-	-	-	-	0.001	***	-	-
	24	0.002	**	-	-	0.001	***	-	-	-	-	-	-	-	-
Combination of aqueous extracts	1	0.002	**	0.01	**	0.01	**	-	-	-	-	-	-	0.002	**
	2	-	-	-	-	-	-	0.005	**	-	-	0.05	*	-	-
	24	-	-	-	-	-	-	-	-	0.02	*	-	-	-	-
O. syriacum ethanolic extract	1	-	-	-	-	-	-	-	-	-	-	-	-	0.04	*
	2	-	-	-	-	-	-	-	-	-	-	0.02	*	-	-
	24	0.001	***	0.001	***	0.05	*	0.002	**	0.001	***	-	-	-	-
C. winterianus ethanolic extract	1	-	-	-	-	-	-	-	-	-	-	0.001	***	0.003	**
	24	0.005	**	0.01	**	0.05	*	0.02	*	0.01	**	-	-	-	-
Combination of ethanolic extracts	2	-	-	-	-	-	-	-	-	-	-	0.05	*	0.005	**
	24	0.005	**	0.005	**	0.04	*	0.01	**	0.003	**	-	-	-	-
O. syriacum EO	1	0.01	**	-	-	-	-	-	-	-	-	-	-	-	-
	2	-	-	0.003	**	-	-	-	-	0.05	*	0.001	***	0.02	*
	4	-	-	-	-	0.02	*	-	-	-	-	-	-	-	-
	24	-	-	-	-	-	-	0.001	***	-	-	-	-	-	-
C. winterianus EO	1	-	-	-	-	-	-	0.001	***	0.002	**	0.05	*	0.02	*
	2	0.04	*	0.005	**	-	-	-	-	-	-	-	-	-	-

	4	-	-	-	-	0.001	***	-	-	-	-	-	-	-	-
Combination of EOs	1	0.02	*	-	-	0.001	***	0.01	**	-	-	-	-	-	-
	2	-	-	-	-	-	-	-	-	0.02	*	-	-	-	-
	4	-	-	0.002	**	-	-	-	-	-	-	0.05	*	0.05	*

EOs: essential oils, S: significance level, ***: $p < 0.001$, **: $p < 0.01$, *: $p < 0.05$, -: not determined.

Table S3. *p*-values and significance levels of the time of the inhibition of biofilm formation of the aqueous and ethanolic *O. syriacum* and *C. winterianus* extracts and their EOs.

Extracts and EOs		Bacterial isolates						Fungal isolates	
		K. pneumonia	S	*S. aureus*	S	*S. intermedius*	S	*C. albicans*	S
Sample	Concentration (mg/mL for the extracts and % for the EOs)	*p*-value (vs. water for the aqueous extracts, ethanol for the ethanolic extracts, and DMSO for the EOs							
O. syriacum aqueous extract	0.3125	-	-	0.001	*	-	-	-	-
C. winterianus aqueous extract	0.625	-	-	-	-	-	-	0.05	*
O. syriacum ethanolic extract	0.3125	0.002	**	-	-	0.001	***	-	-
Combination of ethanolic extracts	5	-	-	0.005	**	-	-	-	-
C. winterianus EO	1.25	-	-	0.01	**	-	-	-	-

EOs: essential oils, DMSO: dimethyl sulfoxide, S: significance level, ***: $p < 0.001$, **: $p < 0.01$, *: $p < 0.05$, -: not determined.

Table S4. *p*-values and significance levels of the time of the destruction of pre-formed biofilms by the aqueous and ethanolic *O. syriacum* and *C. winterianus* extracts.

Extracts and EOs		Bacterial isolates				Fungal isolates			
		K. pneumonia	S	*S. aureus*	S	*C. albicans*	S	*A. niger*	S
Sample	Concentration (mg/mL)	*p*-value (vs. water for the aqueous extracts and ethanol for the ethanolic extracts)							
O. syriacum aqueous extract	0.3125	0.05	*	0.002	**	-	-	-	-
C. winterianus aqueous extract	0.625	-	-	-	-	0.05	*	-	-
Combination of aqueous extracts	0.625	-	-	-	-	-	-	0.001	***
O. syriacum ethanolic extract	5	-	-	-	-	-	-	0.005	**
C. winterianus ethanolic extract	5	-	-	-	-	-	-	0.05	*
Combination of ethanolic extracts	2.5	-	-	-	-	-	-	0.001	***

EOs: essential oils, S: significance, ***: $p < 0.001$, **: $p < 0.01$, *: $p < 0.05$, -: not determined.

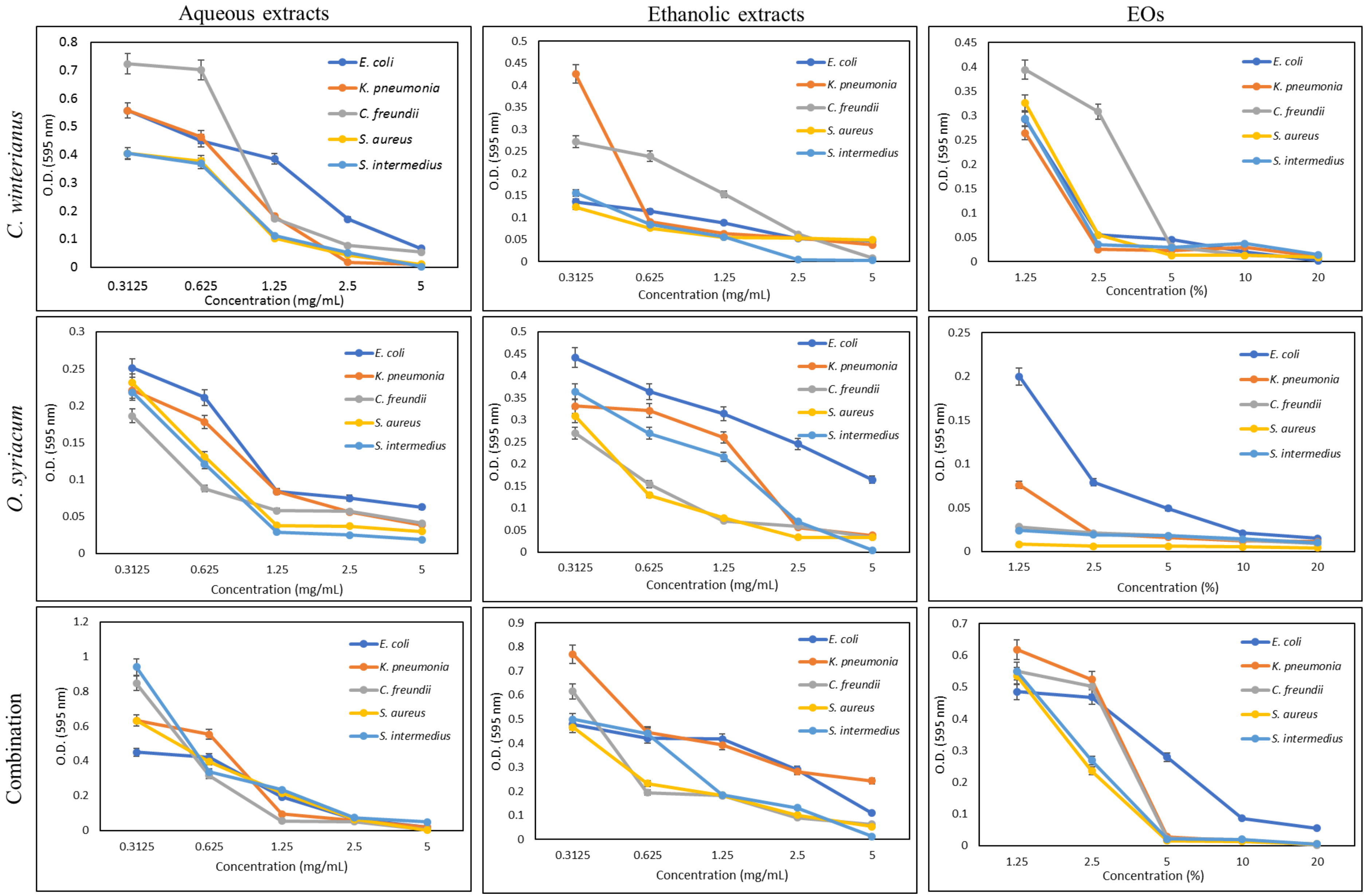

Figure S1. MIC results of the aqueous and ethanolic *O. syriacum* and *C. winterianus* extracts and their EOs against bacterial isolates (EOs: essential oils).

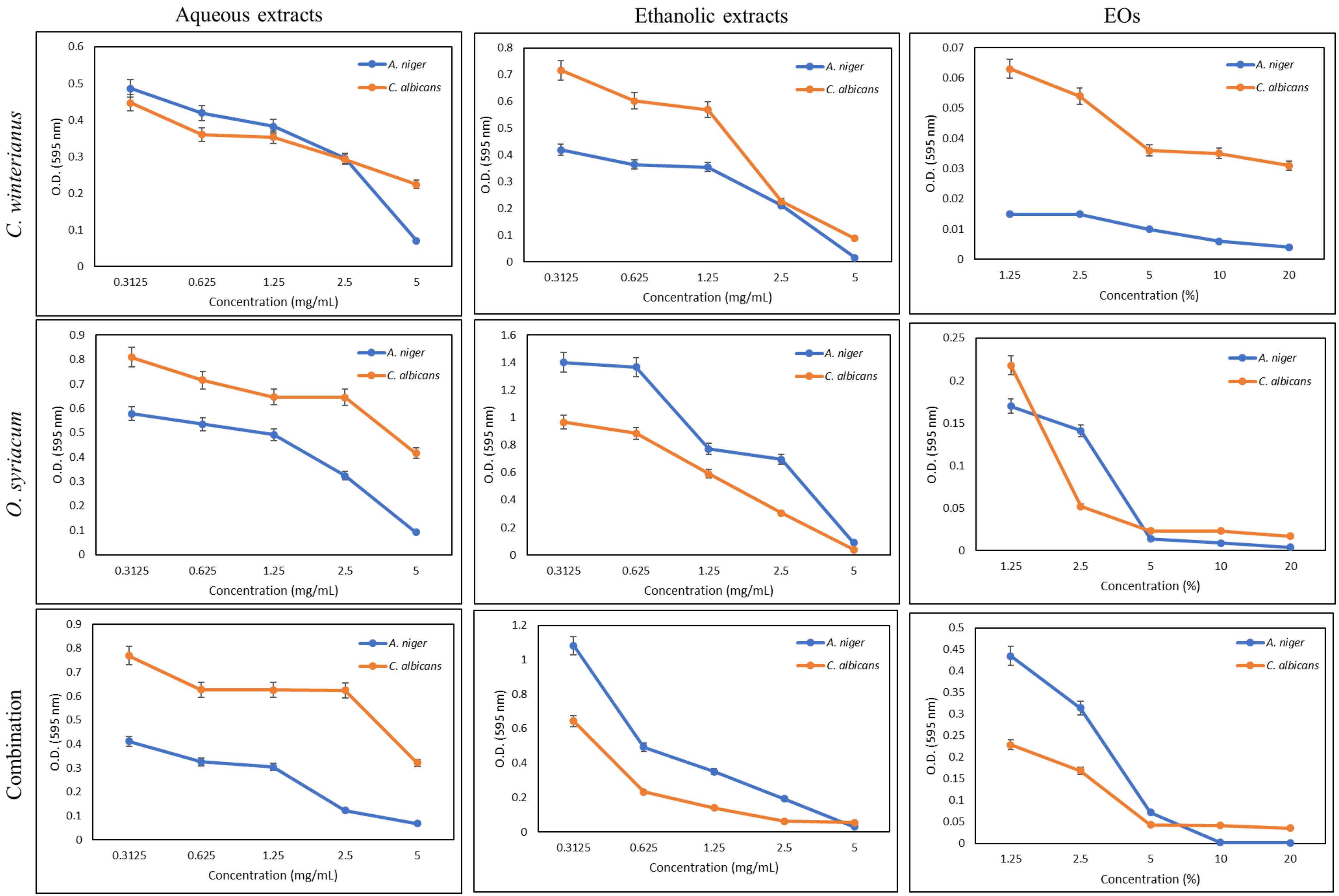

Figure S2. MICs of the aqueous and ethanolic *O. syriacum* and *C. winterianus* extracts, as well as their EOs, against the fungal isolates (EOs: essential oils).

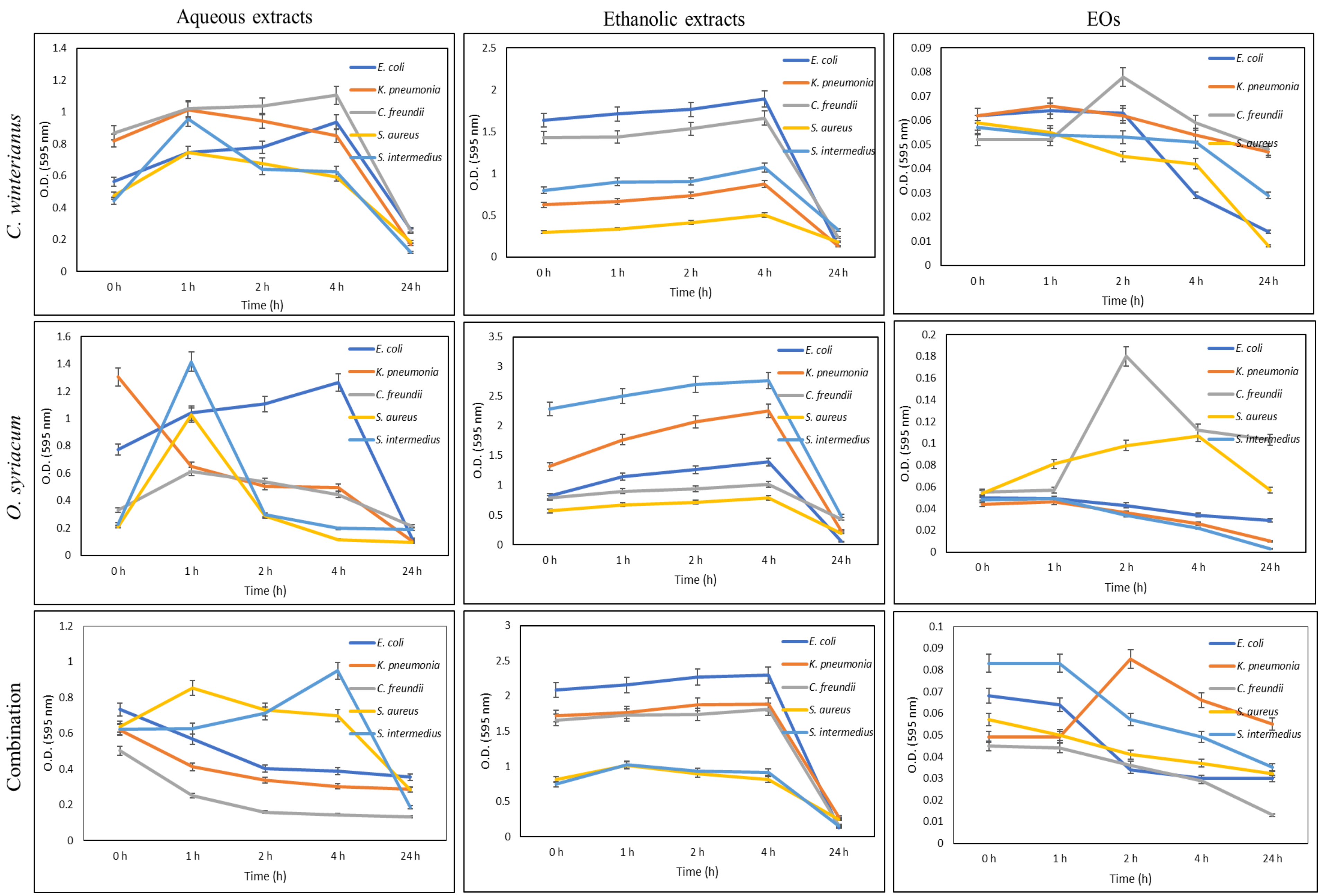

Figure S3. Time kill results of the aqueous and ethanolic *O. syriacum* and *C. winterianus* extracts and their oils against the bacterial isolates (EOs: essential oils).

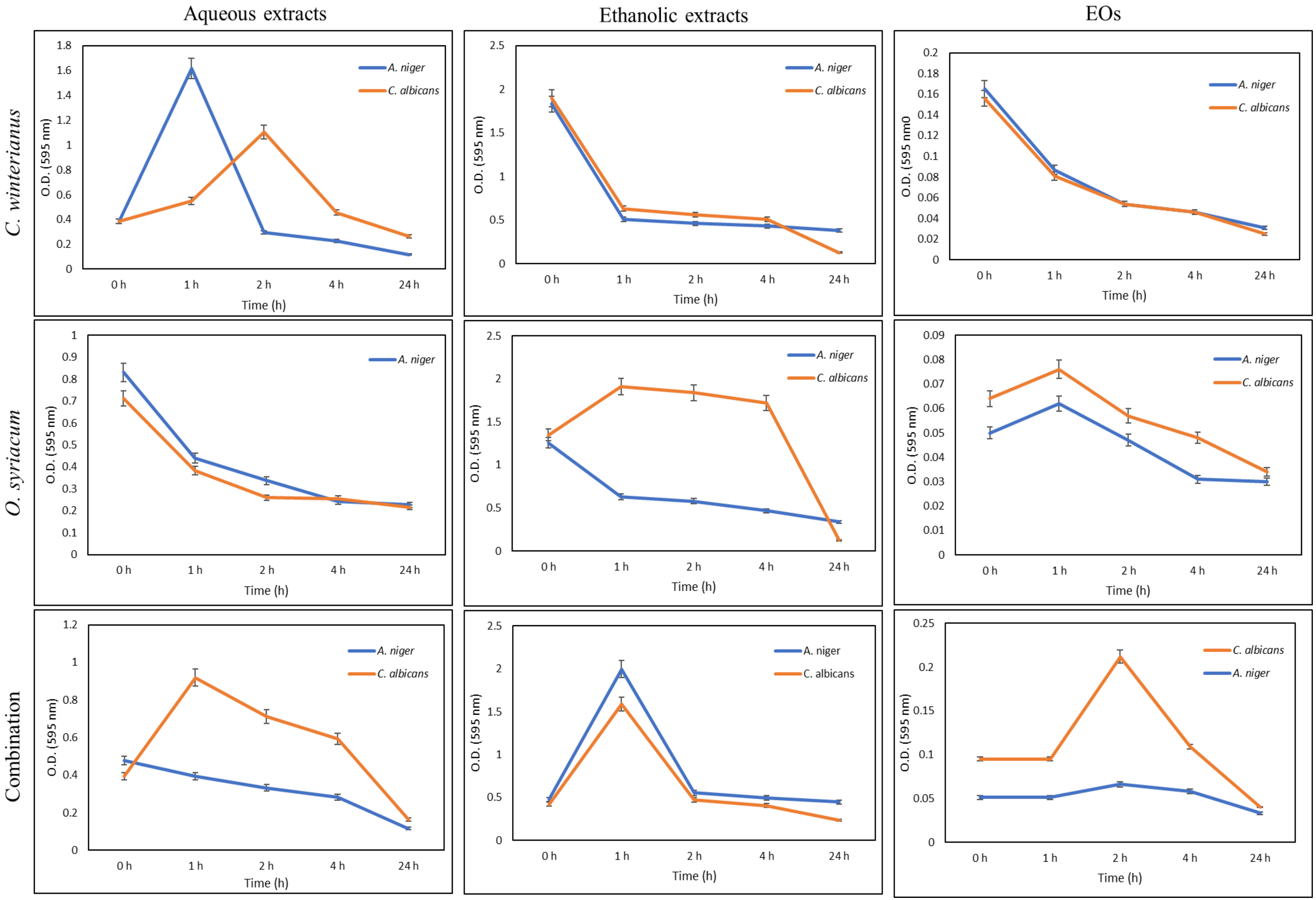

Figure S4. Time kill results of the aqueous and ethanolic *O. syriacum* and *C. winterianus* extracts and their oils against the fungal isolates (EOs: essential oils).
